# Linking warmer nest temperatures to reduced body size in seabird nestlings: possible mitochondrial bioenergetic and proteomic mechanisms

**DOI:** 10.1242/jeb.249880

**Published:** 2025-03-28

**Authors:** Stefania Casagrande, Giacomo Dell'Omo

**Affiliations:** ^1^ Evolutionary Physiology Research Group, Max-Planck-Institut für Biologische Intelligenz 82319, Seewiesen, Germany; ^2^ Ornis italica, 00199 Rome, Italy

**Keywords:** Growth, Metabolic rate, Red blood cells, Heat wave, Mitochondrial bioenergetics, Endotherm

## Abstract

Rapid reduction of body size in populations responding to global warming suggests the involvement of temperature-dependent physiological adjustments during growth, such as mitochondrial alterations in the efficiency of producing metabolic energy, a process that is poorly explored, especially in endotherms. Here, we examined the mitochondrial metabolism and proteomic profile of red blood cells in relation to body size and cellular energetics in nestling shearwaters (*Calonectris diomedea*) developing at different natural temperatures. We found that nestlings of warmer nests had lighter bodies and smaller beaks at fledging. Despite the fact that there was no effect of environmental temperature on cellular metabolic rate, mitochondria had a higher inefficiency in coupling metabolism to allocable energy production, as evidenced by bioenergetic and proteomic analyses. Mitochondrial inefficiency was positively related to cellular stress represented by heat shock proteins, antioxidant enzymes and markers of mitochondrial stress. The observed temperature-related mitochondrial inefficiency was associated with reduced beak size and body mass, and was linked to a downregulation of cellular growth factors and growth promoters determining body size. By analyzing the links between environmental temperature, mitochondrial inefficiency and body size, we discuss the physiological alterations that free-living birds, and probably other endotherms, need to trigger to cope with a warming world.

## Teaser

**Summary:** Temperature-related mitochondrial inefficiency during growth in nestling shearwaters links environmental warming to reduced body size, highlighting physiological adjustments endotherms may require to cope with climate change.

## INTRODUCTION

A reduction in body size is one of the most evident effects of global warming on animals (Cheung et al., 2013; Conradie et al., 2019; Edeline et al., 2013; Gardner et al., 2011; Seebacher et al., 2010; Sheridan and Bickford, 2011; Weeks et al., 2020). Bergmann's rule suggests that smaller endotherms are better adapted to warm environments owing to their high surface area-to-volume ratio, which enhances heat dissipation. However, the rapid shrinkage in body size due to global warming occurs too quickly to be attributed solely to evolutionary processes, suggesting that developmental changes resulting from high temperature exposure play a significant role (Bonamour et al., 2019; Corregidor-Castro et al., 2023; Fuller et al., 2021; Ryding et al., 2021; Sauve et al., 2021). Although indirect effects of environmental warming, such as food and water shortages, have profound and well-documented impacts on growth and body size (Edeline et al., 2013; Youngentob et al., 2021), a direct link with temperature does exist (Andreasson et al., 2018; Andrew et al., 2017; Baudron et al., 2014; Edeline et al., 2013; Gardner et al., 2011; Nord and Giroud, 2020; Nord and Nilsson, 2016). The main physiological candidate underlying this phenomenon is aerobic metabolism (Dillon et al., 2010; Norin and Metcalfe, 2019; Pettersen et al., 2019), which intrinsically scales to temperature following thermodynamic laws (Glazier, 2015; Tattersall et al., 2012). However, how temperature-associated changes in aerobic metabolism affect growth has been studied mostly in ectotherms (Audzijonyte et al., 2019; Barneche et al., 2019; Dawson et al., 2022; Salin et al., 2019; Sheridan and Bickford, 2011). These studies show that higher temperatures increase metabolic rates but reduce growth rates, suggesting that less energy is converted into growth at higher temperatures (Audzijonyte et al., 2019; Barneche et al., 2019; Dawson et al., 2022; Salin et al., 2019; Sheridan and Bickford, 2011). In contrast to ectotherms, endotherms have to allocate energy synthetized by aerobic metabolism (i.e. ATP) to maintain homeothermy (Huey et al., 2012; McKechnie and Wolf, 2019), generating possible trade-offs with growth (Andreasson et al., 2018; Andrew et al., 2017; Cerniglia et al., 1983; Geraert et al., 2005; Mueller et al., 2019; Rodríguez and Barba, 2016). However, how metabolic efficiency in producing energy for growth is altered at high environmental temperatures has hardly been studied in free-living endotherms.

The Scholander–Irving model, which suggests that metabolic rate increases owing to the thermoregulation required to maintain a nearly constant body temperature when environmental temperatures are not optimal, derives its predictions from measuring basal metabolic rate (BMR). By definition, BMR measurements assume that an individual is in a non-growing state. Thus, the model's application to developing individuals has remained unclear. Further challenges to the model arise from the fact that not all endotherms increase their metabolic rate at high temperatures. For example, captive rodents decrease their metabolic rate as temperature rises (Zhao et al., 2022), whereas in birds, species-specific differences in metabolic rate linked to high temperatures have been observed (Albright et al., 2017; Freeman et al., 2022; Levesque and Marshall, 2021; McKechnie et al., 2021; Mitchell et al., 2018). For instance, McKechnie et al. (2021) found significant variation in how arid-zone birds (56 species of different orders) adjust their resting metabolic rate (RMR) beyond the upper limit of their thermoneutral zone, often deviating from the Scholander–Irvine model's predictions. This array of metabolic responses highlights complex and diverse processes to cope with high environment temperatures across endothermic organisms, yet whether and how metabolic trade-offs between thermoregulation and growth occur remain largely unknown.

**Fig. 1.**
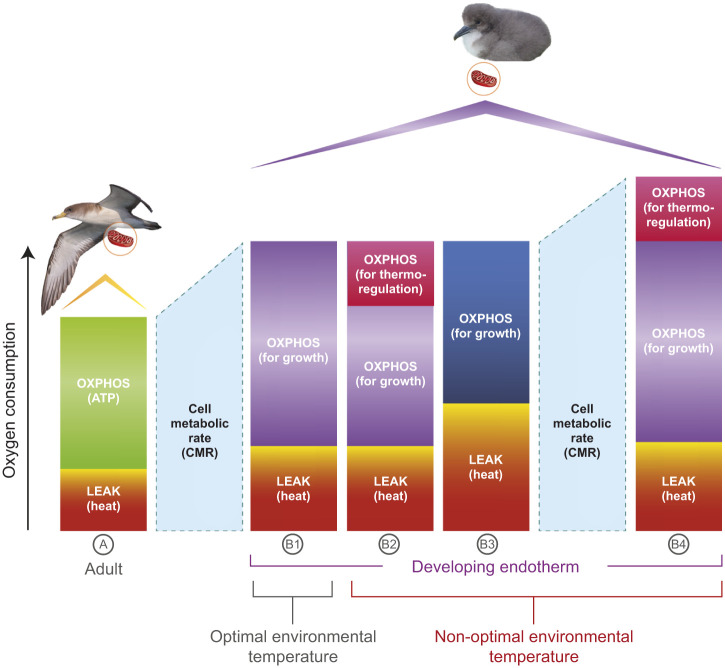
**Simplified representation of the link between oxygen consumption, growth and thermoregulation in relation to the mitochondrial traits measured in the present study.** The cell metabolic rate (CMR, shown in pale blue) consists of two components: oxidative phosphorylation (OXPHOS, which produces ATP for growth and thermoregulation) and proton leak (LEAK, which dissipates energy as heat). The different scenarios illustrate that the same level of oxygen consumption (*y*-axis) can result in varying growth outcomes, such as final body size. In growing individuals (B1–B4), mitochondrial oxygen consumption is higher compared with non-growing birds (A). This increase is primarily driven by the upregulation of OXPHOS (B1), which supports the synthesis of new tissues. In B1, the cost of thermoregulation is negligible because the nestling is developing in optimal thermal conditions. However, a related scenario (B2) occurs when both CMR and OXPHOS remain invariant, but a portion of OXPHOS is reallocated to thermoregulation. In this case, growth is expected to decrease despite the invariance in bioenergetic traits. To assess this scenario, a deeper investigation at the proteome level (growth factors and mitochondrial proteins) would be required. In B3, a similar level of oxygen consumption by the cell results in reduced ATP output owing to a higher proportion of respiration being uncoupled from ATP production, leading to greater heat dissipation (LEAK). Such a state may arise from exposure to high temperatures, which can act as uncouplers. In this scenario, CMR increases, but the proportion of respiration available for growth is diminished compared with the optimal condition in B1. Another potential situation, not depicted here, could involve an elevated CMR with constant growth performance owing to mitochondria being less efficient in coupling respiration with ATP production. In such cases, the mitochondria would need to compensate for ATP production inefficiencies by increasing respiration. [Note: although OXPHOS also generates heat, its contribution is relatively modest compared with the heat generated by proton leak (Rajagopal et al., 2019) and has not been depicted here.]

Linking performance to energetic dynamics in active, free-living animals has been facilitated by the recent validation of measuring mitochondrial metabolism in blood cells as proxy for organismal energy consumptions and needs (Braganza et al., 2020; Casagrande et al., 2023; Liepinsh et al., 2020; Malkoc et al., 2021; Thoral et al., 2024; Tyrrell et al., 2016, 2017). Platelets, lymphocytes, monocytes and non-mammalian red blood cells (RBCs) possess functional active mitochondria that respond to changes in organismal energetic needs and metabolic rate (Braganza et al., 2020). For instance, in pied flycatcher (*Ficedula hypoleuca*) females, blood mitochondrial metabolism increased from incubation to chick rearing, to match the energetic requirements of specific life-history stages (Stier et al., 2019). Cell metabolic rate of great tits (*Parus major*) decreased when individuals shifted from an active to a resting state (Malkoc et al., 2021), or increased in different species of passerines to match the higher energy expenditure associated with thermoregulation (Nord et al., 2021). Although the energetic costs of thermoregulation are predominantly managed by other tissues, such as skeletal muscle or, when present, brown adipose tissue, blood cells can integrate signals from across the organism, reflecting metabolic adjustments to environmental and internal demands (Braganza et al., 2020). Measuring oxygen consumption in blood cells offers the possibility to measure respiration rates in the mitochondria, where aerobic metabolism actually occurs (Koch et al., 2021; Lane and Martin, 2010). The mitochondria supply the majority of the organismal energy currency (ATP) through the process of oxidative phosphorylation (OXPHOS; Fig. 1). Distinguishing distinct mitochondrial metabolic traits can reveal imbalances in the efficiency of producing ATP, which may have major consequences for processes such as growth (Fig. 1). So far, we know of only two studies (Ton et al., 2021; Udino et al., 2021) that have analyzed mitochondrial metabolism in endotherms in relation to body size and warm temperatures. These studies on captive zebra finch (*Taeniopygia guttata*) nestlings showed that high nest temperature enhanced mitochondrial metabolic rates and reduced mitochondrial efficiency in producing ATP, but without affecting body size because the increase in metabolic rate offset mitochondrial inefficiency (Ton et al., 2021; Udino et al., 2021). However, it is unclear whether these results can be generalized because the ratio between body volume and surface area in these small passerines favors the dissipation of heat when compared with larger animals (Huey et al., 2012; McKechnie and Wolf, 2019; Tattersall et al., 2012). In addition, lab conditions, where food and water are provided without limitation, could have eliminated the trade-off between energy allocation for growth versus thermoregulation.

To investigate which metabolic adjustments mediate changes in body size in response to temperature, we explored links between nest temperature and RBC mitochondrial metabolism on body size (mass and beak size) in nestlings of Scopoli's shearwater, *Calonectris diomedea* (shearwater hereafter), breeding on Linosa island, Italy. To gain insights into possible cellular mechanisms underlying this link, we analyzed the RBC proteome, focusing on parameters related to growth regulation, mitochondrial ATP production and cellular stress. We focused on the proteome of RBCs for our investigations because blood has been recognized as an effective ‘sentinel’ tissue that can express up to 80% of the genes that are typically expressed in specific tissues (defined as ‘illegitimate expression’), thereby providing a comprehensive overview of the body's physiological processes (Liew et al., 2006).

In this study area, it is possible to investigate the effect of environmental temperature mainly without the interference of other weather variables, such as precipitation (Edeline et al., 2013), which are rare events during the breeding season (e.g. no rain was observed during the entire breeding season in our study). Each breeding pair produces a single egg per year, which creates naturally standardized brood sizes. From the first week after hatching, shearwater chicks spend the entire daytime alone in the nest, making it possible to sample them without disrupting parental care. Moreover, adults feed chicks with fish oil in a single bout and at night, when temperatures are mild, minimizing the negative effect of high temperature on feeding rate (Cerniglia et al., 1983). Like other avian offspring, they are able to thermoregulate around 1–3 weeks after hatching (Walter and Seebacher, 2009).

In general, we hypothesize that warmer nest temperatures during development negatively affect nestling growth and mitochondrial efficiency, potentially leading to downstream effects on cellular metabolism and stress markers. One hypothesis is that when exposed to elevated nest temperatures, nestlings are unable to further increase their metabolic rate because they are already close to their maximum capacity owing to the high energy demands of growth (Fig. 1, B2). In this case, a portion of the OXPHOS capacity would need to be reallocated to support thermoregulation, resulting in a reduced availability of energy for growth processes (Fig. 1, B2). Another possibility is that nestlings do not efficiently thermoregulate, and elevated nest temperatures increase mitochondrial inefficiency (Fig. 1, B3). This inefficiency may increase metabolic costs, affecting the allocation of energy toward growth (Fig. 1, B4), leading to measurable physiological and molecular consequences (see question 5 below). An alternative hypothesis is that, although nestlings are expected to have a higher metabolic rate than adults (Fig. 1, A), they are further able to increase aerobic metabolism to match the demands of active thermoregulation without compromising their growth (Fig. 1, B3). Based on these general hypotheses, we outlined specific questions underlying the following predictions. (1) Body size: nestlings that develop in warmer nests will exhibit smaller body size at fledging. (2) Mitochondrial function: nestlings exposed to warmer temperatures will show higher mitochondrial inefficiency in coupling respiration with ATP synthesis (higher LEAK respiration/lower OXPHOS respiration, hereafter referred to as ‘mitochondrial inefficiency’). (3) Cell metabolic rate (CMR), a proxy for organismal metabolic rate: CMR will increase with nest temperature owing to higher thermoregulatory costs (Fig. 1, B4) or to compensate for higher LEAK respiration (Fig. 1, B3; Udino et al., 2021). (4) Growth–mitochondria link: body size traits will be negatively associated with mitochondrial inefficiency. (5) Molecular pathways: temperature-related mitochondria inefficiency will be associated with: (i) lower levels of proteins related to cell proliferation and cell growth (specifically, growth factors, mechanistic target of rapamycin (mTOR) signaling, and downstream growth promoters); (ii) lower levels of proteins related to ATP production [specifically, glycolysis, a cytoplasmic metabolic pathway that breaks down glucose to produce ATP and NADH; β-oxidation, a metabolic process in which fatty acids are broken down in the mitochondrial matrix to generate acetyl-CoA, NADH and FADH_2_ for oxidative phosphorylation; the TCA cycle, mitochondrial matrix processes generating electron donors (NADH and FADH_2_) for oxidative phosphorylation; and oxidative phosphorylation, the process in which ATP is synthesized using energy released by electrons transferred through the electron transport chain]; (iii) higher levels of AMP kinase (AMPK), which is upregulated when ATP levels are low; and (iv) higher levels of markers of cell stress [specifically, heat shock proteins (HSP), response to oxidative stress, and markers of mitochondrial stress].

## MATERIALS AND METHODS

### Ethical statement

All the experimental procedures described in the study were granted by the authorities of Regione Sicilia (protocol no. 24156) and approved by Istituto Superiore per la Protezione e Ricerca Ambientale (ISPRA, protocol no. 24065).

### Model species, study area and experimental design

**Fig. 2.**
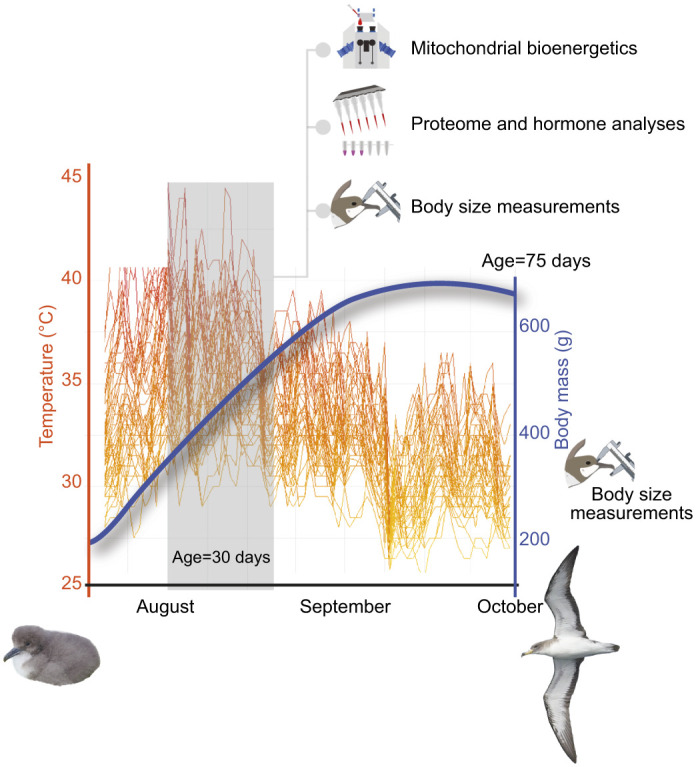
**Maximum nest temperatures reported for each day from hatching to fledging for different nests (lines).** The blue curve charts the body mass trajectory of the nestlings. The gray shaded region indicates the month of sampling, which coincides with the year's warmest period and steepest part of chick growth.

The study was conducted in 2021 on Linosa, Italy (35°51′33.05″N, 12°51′45.79″E), a volcanic island of 5.43 km^2^ located in the Sicilian channel that hosts several thousand breeding pairs of Scopoli's shearwater [*Calonectris diomedea* (Scopoli 1769)]. Nests are highly variable in temperature depending on their vicinity to the sea, shade from vegetation or depth below rocks, a condition setting that allowed us to evaluate the effect of naturally varying temperature on chick development. It should be noted that 2021 was characterized by an intense and prolonged heatwave in July–August (named ‘Lucifer’) that affected the study area during the steepest part of shearwaters’ growth (Fig. 2; https://climate.copernicus.eu/europe-experienced-its-warmest-summer-record-2021-accompanied-severe-floods-western-europe-and-dry).

### Nest temperature recording

Nest temperature was recorded hourly by placing a temperature logger (Thermochron iButton DS1921G, Analog Devices Inc., Louisville, KY, USA; temperature range from –30°C to 85°C, resolution of 0.5°C) into each nest. Temperature loggers were positioned in the nests during the pre-hatching monitoring in mid-July. They were taped to a small stone placed into the nest in a way to avoid both contact of the logger with bird's bodies and direct sun exposure.

The mean nest temperature (average of the temperatures recorded over 24 h, averaged for nests and months) from hatching to fledging for the 47 focal nests was 30.15±0.14°C (range 28.44–32.34°C). The maximum average temperature was 35.28±0.29°C (range 31.83–39.77°C, averaged for nests and months) and the minimum average temperature was 25.28±0.15°C (averaged for nests and months). Narrowing the time frame to the hottest days of the study year (i.e. 20 July–20 August 2021), which overlap with the steepest part of the grow curve (Fig. 2), the average nest temperatures recorded during the daytime (from 08:00 to 20:00 h) were: mean 31.92±0.22°C (range 28.86–34.87°C), maximum 37.54±0.41°C (range 32.0–43.0°C) and minimum 26.27±0.16°C (range 23.5–28.5°C). The average nest temperature recorded in the morning, 1 h before sampling (i.e. ‘nest temperature’), was 30.75±0.22°C (range 28.0–34.0°C). Nest temperature was used in models run to explain the variation of physiological variables measured in the blood and the variation of body size traits. Nest temperatures measured in the morning were indeed correlated with the mean and maximum temperatures recorded during the day in the hottest days of the nesting cycle (morning temperature–average temperature: *r*=0.45, *P*=0.001; morning temperature–maximum temperature: *r*=0.35, *P*=0.016). Average maximum temperature was on average 7.65°C higher than average morning temperature. Morning temperature was correlated also with the numbers of hours exceeding 35°C (*r*=0.40, *P*=0.004) and with average temperature recorded during the whole nest cycle (*r*=0.27, *P*=0.02). The comparison of models run to analyze the variation of body size with temperature indicated that models with nest temperature recorded in the morning showed a better fit than all the other models for both beak size and body mass (see Table S1 for comparison of models run with different temperatures). This is not surprising because in diurnal birds, metabolic settings follow a diel rhythm that can anticipate the upcoming conditions by responding to morning temperatures (Pessato et al., 2023).

### Sampling

Blood samples were taken in mid-August, which is the hottest time of the year in the study area (Fig. 2). As the nesting cycle is quite synchronous in this population, with all laying and hatching events occurring within a 2-week window (Fig. 2), average nestling age at sampling was 23.55±0.18 days. This is also the age at which they have the fastest growth rates (Fig. 2). Nests were visited in the morning (08:00–10:00 h), and a 200 µl blood sample was taken from the tarsal vein using a heparinized syringe, within 4 min of nest contact (mean: 179±11.59 s). Blood was immediately transferred into a heparinized 0.5 ml tube and kept in a cooled bag until mitochondrial analysis. To minimize the time elapsed between blood collection and analysis, we sampled a maximum of six birds per day. To separate RBCs, blood was centrifuged immediately after fieldwork, by processing two samples at a time and in the same order of collection. Mitochondrial analyses took 30 min for every two samples – which were run in parallel – allowing a maximum of 5 h to elapse from the beginning of sample collection to the end of mitochondrial analysis. After measuring the length and depth of the beak (caliper ±0.1 mm) and the body mass (Pesola scale ±5 g), nestlings were immediately returned into their nest. Bill measurements and body mass were taken a second time just prior to fledging when nestlings were about 11 weeks old (75±0.46 days after hatching, range 71–84 days; Fig. 2). Four of the 51 initially sampled nestlings could not be re-measured at day 75 because they died in the meantime from starvation following nest abandonment by parents (2 chicks), predation (1 chick) or disappearance for unknown causes (1 chick). Consequently, all results refer to the remaining 47 nestlings.

### Mitochondrial bioenergetics

Oxygen consumed by aerobic metabolism during mitochondrial respiration was measured in RBCs (Casagrande et al., 2020; Stier et al., 2017) with a Clark electrode high resolution respirometer chamber (Oxygraph-2k, Oroboros Instruments, Innsbruck, Austria). We chose an assay temperature of 40°C in accordance with what is known for adults of diverse species of procellariforms in normothermic phase (average=39.4°C; range: 37.5–41°C; McKechnie, 2022). This was then confirmed by cloacal temperature in the following year (range: 36.3–41.6°C). The protocol has already been published (Stier et al., 2017), thus a full description is reported in the Supplementary Materials and Methods. Mitochondrial respiration was quantified as the O_2_ consumed by intact cells at the following stages (Fig. S1): (1) cellular metabolic rate (ROUTINE, hereafter referred to as CMR), the basal respiration of the cells in their endogenous state; (2) proton leak (LEAK), a residual respiration that is not coupled to ATP synthesis and that releases heat (also called ‘uncoupled respiration’ or ‘non-phosphorylating respiration’; Fig. S1), measured by inhibiting ATP synthase through the addition of 1 µg ml^−1^ of oligomycin; and (3) the remaining basal respiration that is affected by oligomycin provides the quantification of oxidative phosphorylation (OXPHOS), the process through which ATP is produced (Fig. S1). We also measured (4) functionality of the electron transport system (ETS), by adding the mitochondrial uncoupler carbonyl cyanide m-chlorophenyl hydrazine (CCCP mitochondria: 2 to 3 titrations of 1 µmol l^−1^ aliquots) (Fig. S1). Basal respiration rates are restricted by the ATP demand of the cell, whereas the mitochondrial uncoupler causes the flow of electrons through the ETS to be independent of the transformation of ADP into ATP. This condition is a reliable indication of the state of the ETS and of the capacity of transport and oxidize energy substrates (Divakaruni and Jastroch, 2022). It represents a suitable variable for internal standardization of respiratory adjustments. All traits were corrected for non-mitochondrial O_2_ consumption by adding antimycin A (5 µmol l^−1^), an inhibitor of mitochondrial complex III (Fig. S1). All measures were normalized by the volume of RBCs (average: 37.38 µl; range: 30–40 µl) used for the analysis after assuring a linear dependence of oxygen consumption rate on RBC volume (*F*_1,49_=85.12, *P*<0.0001) and expressed as pmol O_2_ min^−1^ ml^−1^ of RBCs. From these measurements we calculated mitochondrial inefficiency in two ways, specifically as the ratio of LEAK to CMR (FCR_LEAK/CMR_) and ETS (FCR_LEAK/ETS_).

### Proteome analysis

The untargeted proteome was analyzed through liquid chromatography mass spectrometry (LC-MS) by the Max Planck Institute of Biochemistry. All the details of the procedure can be found in the Supplementary Materials and Methods.

### Molecular sex determination

Because males tend to be bigger than females, even during development, and metabolic rate scales with body mass, we took this information into account. Nestling sex was determined from blood using a molecular protocol validated for the model species (Tagliavia et al., 2023) and is described in the Supplementary Materials and Methods.

### Relationship between body temperature and nest temperature

In 2022, we measured the body temperature of chicks hatched in the same nests monitored in 2021. This was important to interpret the results on metabolism. Specifically, we recorded the cloacal temperature of chicks twice, once in the morning, between 08:00 and 10:00 h, and once in the afternoon, between 16:00 and 17:00 h, when nest temperature was peaking. Cloacal temperature was measured using a portable thermometer BIO-TK8851 provided with a probe with an isolated sensor (BIO_BRET2_ISO) having a resolution of 0.1°C (Bioseb, FL, USA). Nest temperature was recorded following the 2021 protocol described above.

### Statistics and hypothesis testing

For our statistical analysis, we utilized the R software environment (R version 4.3.1; https://www.r-project.org/) using the linear modeling function ‘lm’. Graphical representations were generated using the ‘ggplot2’ package in R. All mean values are presented ±s.e.m.

#### Procedure to test questions 1–4

After checking whether variables met the assumptions of homogeneity of variance and normal distribution by visually analyzing the graphical distributions of fitted values versus their residuals, protein concentrations were log transformed to meet these standards. Statistical significance was determined by *P*-values (α=0.05) for each fixed effect together with a measure of effect size (Cohen's effect size *f*^2^ for multiple linear regression model) calculated for each model (small: *f*^2^=0.05; medium: *f*^2^=0.15; large: *f*^2^=0.35).

The model used to investigate the effect of temperature on body mass and beak size at fledgling (question 1) included nest temperature as predictor, while accounting for the sex of the chick. Beak size was described by the first principal component (PC1) generated using standardized values of beak length and beak depth (Pearson's correlation: *r*=0.71, *P*<0.0001). PC1 of beak measured at independence represented the 73.07% of trait variation. In a further analysis, we added initial values of the respective body size trait to account for initial conditions, but the results did not change substantially (Table 1).

**Table 1.** Effects of nest temperature on body size (question 1)

Variable (*R*^2^, *f*^2^)	Estimate±s.e.	*t*_1,44_	*P*
Beak size (0.57, 1.32)			
Nest temperature	−0.28±0.08	−3.39	0.0015
Sex (male)	1.54±0.25	6.15	<0.0001
Body mass (0.27, 0.37)			
Nest temperature	−17.79±6.30	−2.83	0.007
Sex (male)	46.56±19.35	2.41	0.02
Beak size (0.70, 2.33)			
Nest temperature	−0.38±0.11	−3.69	0.0006
Sex (male)	0.92±0.19	0.25	0.0008
Initial beak size	0.47±0.13	4.35	<0.0001
Body mass (0.60, 1.50)			
Nest temperature	−21.34±9.48	−2.25	0.029
Sex (male)	30.32±20.62	1.47	0.1488
Initial body mass	0.32±0.17	1.88	0.067

Values in brackets after the dependent variable are *R*^2^ and Cohen's effect size.

To ensure that nest temperature was not indicative of broader nest or parental quality – i.e. that the choice of a non-optimal nest by inexperienced parents did not indirectly affect body size – we analyzed data from the previous year when no heat waves occurred (unfortunately, we did not have thermocouples then). By including body size data from the same nests monitored the previous year, we ran a model for each body size trait, including nest as a random factor (pairs remain faithful to the nest; data not shown). The analysis excluded the possibility of an indirect effect of nest identity (nest variance component for beak size: 0.22 [95% CI: −2.48, 2.93]; nest variance component for body mass: 0.12 [95% CI: −0.11, 0.36]). Further support came from the lack of differences in body size of chicks in the early stages of growth (see Results).

To understand how mitochondrial metabolism was related to nest temperature (questions 2 and 3), we ran one model for each of the mitochondrial traits considered as a response variable (i.e. CMR or ROUTINE, OXPHOS, LEAK, ETS, FCR_LEAK/CMR_, FCR_LEAK/ETS_) and nest temperature as predictor, while correcting for sex of the chick and duration of the blood sampling (measured in seconds from the first approach to the nest to the end of the bleeding; Table 2).

**Table 2.** Effects of nest temperature on mitochondria traits (questions 2 and 4)

Variable (*R*^2^, *f*^2^)	Estimate±s.e.	*t*_1,44_	*P*
CMR (0.05, 0.04)			
Nest temperature	−15.31±10.19	−1.02	0.317
Sex (male)	37.95±31.15	−1.22	0.230
Sampling duration	0.03±0.19	0.14	0.890
OXPHOS (0.13, 0.15)			
Nest temperature	−21.10±9.52	−2.22	0.032
Sex (male)	33.68±29.11	−1.15	0.254
Sampling duration	−0.16±0.17	−0.89	0.379
LEAK (0.15, 0.18)			
Nest temperature	10.79±5.20	2.07	0.040
Sex (male)	4.27±15.91	−0.27	0.790
Sampling duration	0.18±0.10	1.90	0.064
ETS (0.05, 0.05)			
Nest temperature	−25.04±16.92	−1.48	0.146
Sex (male)	5.20±34.84	−0.15	0.882
Sampling duration	0.09±0.21	0.41	0.680
FCR_LEAK/CMR_ (0.18, 0.22)			
Nest temperature	0.04±0.02	2.59	0.013
Sex (male)	0.05±0.04	1.02	0.312
Sampling duration	0.0005±0.0003	1.74	0.089
FCR_LEAK/ETS_ (0.25, 0.33)			
Nest temperature	0.04±0.01	3.36	0.0017
Sex (male)	0.002±0.03	0.07	0.945
Sampling duration	0.0004±0.0.002	1.83	0.073

Values in brackets after the dependent variable are *R*^2^ and Cohen's effect size. CMR, cell metabolic rate; OXPHOS, oxidative phosphorylation; ETS, electron transport system functionality; FCR_LEAK/ETS_, flux control ratio calculated as the ration between proton leak and the maximal capacity of the electron transport chain.

To investigate whether the physiological variables that responded to nest temperature explained body size at fledging (question 4), we ran one model for each body size trait, i.e. beak size (PC1) and body mass, using mitochondria bioenergetics traits in turn as predictors, while accounting for the sex of the chick (Table 3). Nest temperature was not included in these models because of the strong evidence that it was associated with the metabolic covariates included (see related results). Thus, including nest temperature and temperature-associated metabolic traits would have caused collinearity issues.

**Table 3.** Effects of temperature-mediated mitochondrial inefficiency on body size traits at fledging with and without correction for starting body size values (question 3)

Variable (*R*^2^, *f*^2^)	Estimate±s.e.	*t*_1,44_	*P*
Beak size (0.57, 1.32)			
FCR_LEAK/ETS_	−3.22±0.95	−3.39	0.0015
Sex (male)	1.64±0.25	6.65	<0.0001
Beak size (0.71, 2.35)			
FCR_LEAK/ETS_	−3.09±0.79	−3.92	0.0003
Sex (male)	0.99±0.25	3.98	0.0003
Initial beak size	0.48±0.11	4.57	0.000004
Body mass (0.25, 0.34)			
FCR_LEAK/ETS_	−189.43±74.24	−2.55	0.014
Sex (male)	53.43±19.27	2.77	0.008
Body mass (0.36, 0.56)			
FCR_LEAK/ETS_	−191±69.34	−2.76	0.0084
Sex (male)	29.00±20.11	1.44	0.16
Initial body mass	0.43±0.16	2.73	0.0091

Values in brackets after the dependent variable are *R*^2^ and Cohen's effect size.

In an initial stage, we considered whether the effect was sex-dependent by including the interaction term sex×predictor of interest in all models. However, this interaction was never significant and was excluded from the final analyses [results not shown, but see data in Dryad (Casagrande and Dell'Omo, 2025): doi:10.5061/dryad.8931zcs03]).

To test question 5, we employed linear regression modeling to evaluate the relationship between mitochondrial inefficiency (FCR_LEAK/ETS_, which was the variable that was most strongly associated with nest temperature and with body size traits) and log(10+0.01)-transformed expression levels of proteins detected in RBCs by the untargeted proteomic analysis. The regression analysis showed that mitochondrial inefficiency was significantly associated with 1012 out of the 3758 detected proteins. Here, we present the proteins that were related to question 5. The physiological function of each protein was checked on www.uniprot.org or www.genecards.org in order to detect those related to growth (i.e. growth factors, mTOR and growth promoters or effectors), mitochondrial oxidative phosphorylation (subunits of complex I, II, III, IV and V), other processes related to ATP production (glycolysis, TCA cycle, β-oxidation), ATP levels (AMP kinase), heat stress (heat shock proteins), response to oxidative stress (antioxidant enzymes) and mitochondrial stress response [see specific proteins in Figs 5 and 6, Table S3 and data in Dryad (Casagrande and Dell'Omo, 2025): doi:10.5061/dryad.8931zcs03]). For the proteome analysis, effect sizes with 95% confidence intervals7 that did not overlap zero were considered statistically meaningful and results were visualized by forest plots.

To investigate the relationship between body temperature and nest temperature in the following year, we conducted linear regression models with body temperature as the dependent variable, and nest temperature and time of day (two levels: morning and afternoon) as predictors. Individual identity was included as a random factor to account for repeated measures of body temperature taken at two times of the day. To compare nest temperatures between 2021 and 2022, we used nest temperature as the dependent variable, time of the day as the predictor and nest as a random factor to account for repeated measures within each nest.

## RESULTS

### Nest temperature, body size and mitochondrial performance

#### Question 1

**Fig. 3.**
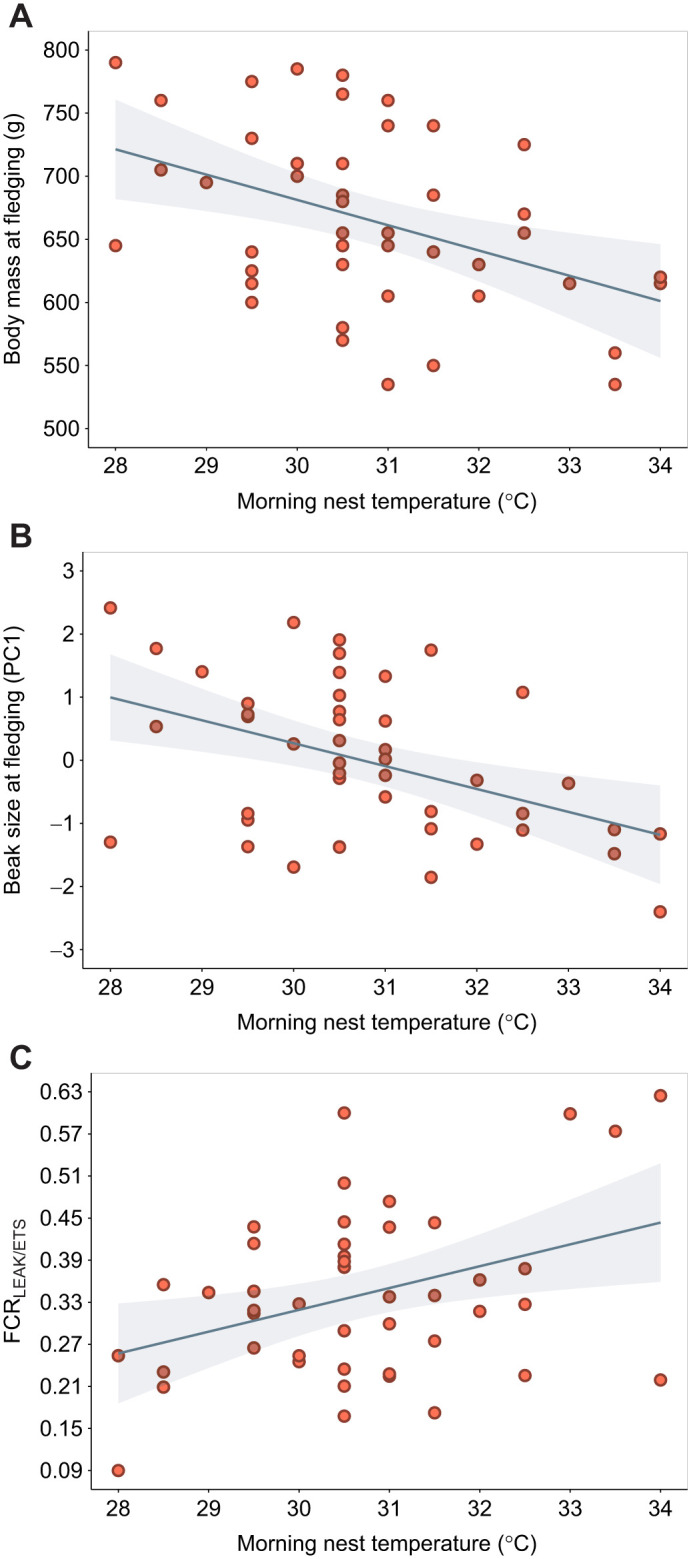
**Association between nest temperature, body mass, beak size and mitochondrial inefficiency.** Effect of morning nest temperature on (A) body mass, (B) beak size (PC1 of beak depth and length) and (C) mitochondrial inefficiency in producing ATP (flux ratio FCR_LEAK/ETS_).

Nestlings that developed in warmer nests had a lower body mass at fledging and a smaller beak (Table 1, Fig. 3A,B). The effect of temperature on beak size and body mass was not present at first sampling (about 4 weeks post-hatching; beak: β_nest temperature_=−0.05±0.09, *t*=−0.49, *P*=0.62; β_sex_=1.32±0.29, *t*=4.47, *P*<0.0001, *R*^2^=0.33; body mass: β_nest temperature_=–10.75±5.47, *t*=−1.967, *P*= 0.06; β_sex_=51.19±16.71, *t*=3.06, *P*=0.004, *R*^2^=0.26).

#### Questions 2 and 3

Nestlings that developed in warmer nests showed a lower OXPHOS and a higher LEAK, which led to a higher mitochondrial inefficiency in coupling oxygen consumption with ATP synthesis (FCR_LEAK/CMR_ and FCR_LEAK/ETS_; Table 2, Fig. 3C). There was no evidence that nest temperature affected CMR or maximal capacity of the ETS (Table 2). See Fig. S2 for a visualization of mitochondrial bioenergetic traits.

### Relationship between thermal-associated mitochondrial inefficiency and body size (question 4)

**Fig. 4.**
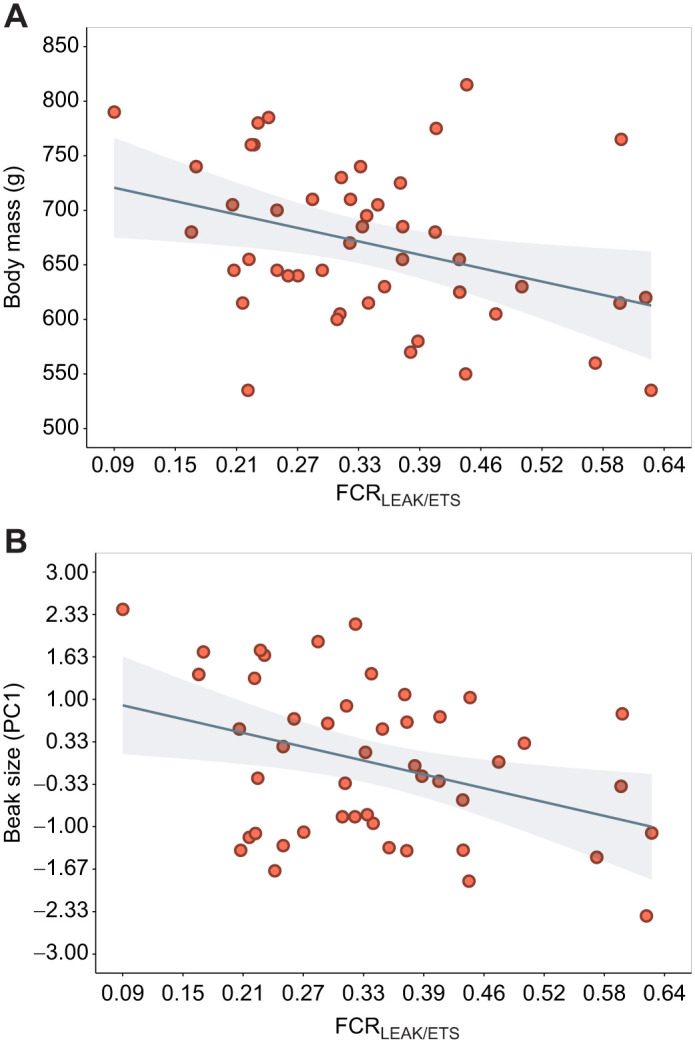
**Effect of mitochondrial inefficiency on body mass and beak size.** Relationship between mitochondrial inefficiency expressed by the flux ratio FCR_LEAK/ETS_ and (A) body mass and (B) beak size (PC1 of beak depth and length) measured at fledging.

Body size traits were better explained by FCR_LEAK/ETS_ in comparison with all the other mitochondrial traits that varied with nest temperature (Table S2 for model comparison). The higher mitochondrial inefficiency FCR_LEAK/ETS_ observed in warmer nests was associated with smaller beak size and lighter body mass (Table 3, Fig. 4A,B). See Table S2 for further results on FCR_LEAK/CMR_, OXPHOS and LEAK.

### Proteome analysis (question 5)

The full name of all proteins is reported in Table S3. Functions of specific proteins are described in the Discussion.

#### Cell proliferation and growth

**Fig. 5.**
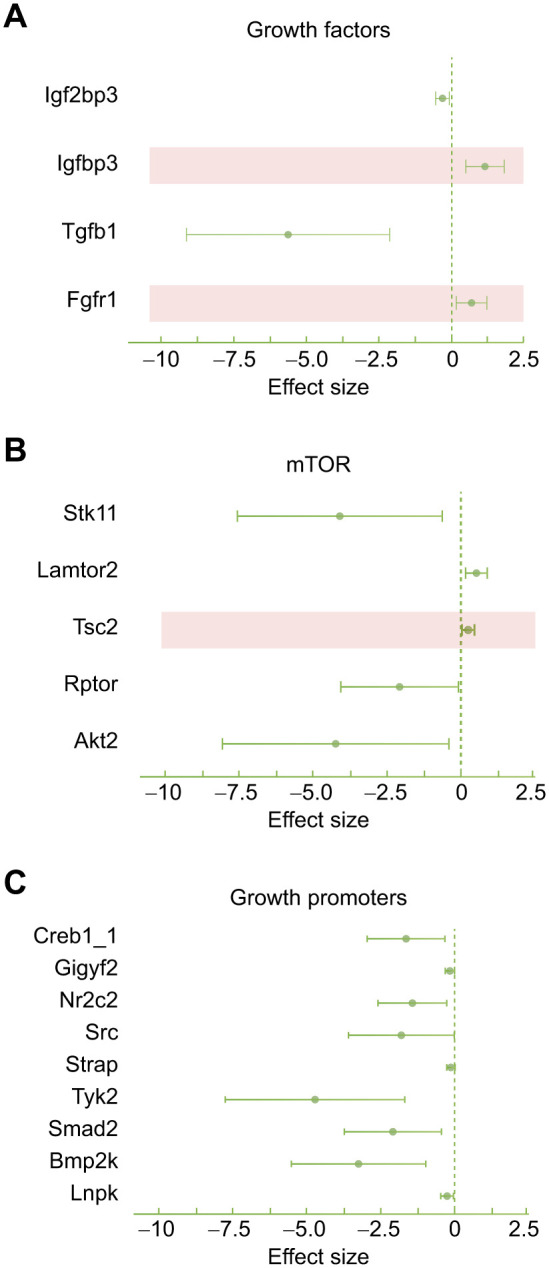
**Effect sizes represented by the regression estimates between mitochondrial inefficiency and growth factors, mTOR and growth promoters.** (A) Growth factors; red shading indicates proteins with inhibitory effect on growth. (B) mTOR; red shading indicates a protein with an inhibitory effect on mTOR. (C) Growth promoters or effectors (full name of proteins provided in Table S3).

The regression analysis of the proteome detected 18 proteins related to the regulation of growth that were related to the mitochondrial inefficiency associated with variations in nest temperature, as follows. (a) Growth factors: mitochondrial inefficiency was negatively associated with the growth promoters and positively related to the growth suppressors found (Fig. 5A; Table S3). (b) mTOR: mitochondrial inefficiency was associated with proteins related to mTOR, which controls growth and metabolism in relation to cellular energy reserves (Martin and Hall, 2005; Valvezan and Manning, 2019). Specifically, mitochondrial inefficiency was negatively associated with the mTOR activators, whereas it was positively associated with the mTOR suppressor TSC2 (Fig. 5B; see also Table S3). (c) Growth promoters: high temperature-associated mitochondrial inefficiency was negatively related to all downstream growth promoters found in the proteome (Fig. 5C, Table S3). Overall, these results indicate that the biochemical processes promoting cell growth, upon which whole-organism growth is based, were diminished in nestlings with high mitochondrial inefficiency.

#### ATP availability

**Fig. 6.**
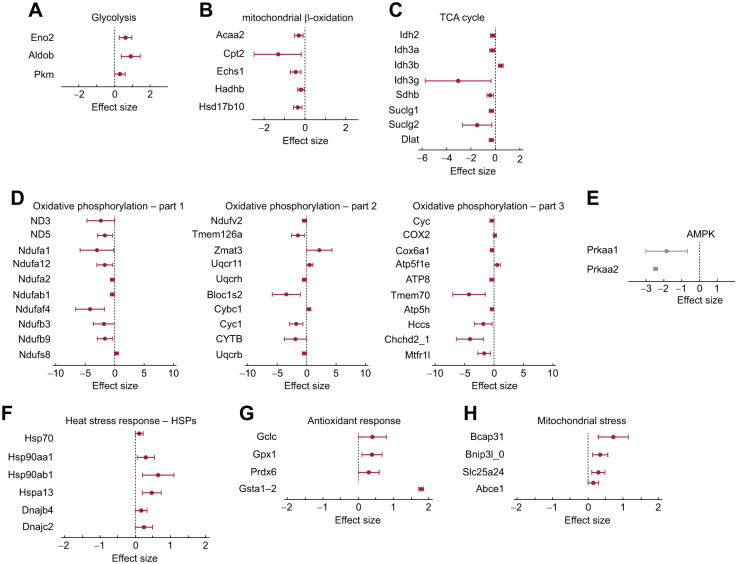
**Effect sizes represented by the regression estimates between mitochondrial inefficiency and protein subunits of the following.** (A) Glycolysis, (B) β-oxidation, (C) TCA cycle, (D) the mitochondrial electron transport system involved in oxidative phosphorylation, (E) AMP-kinase, (F) heat shock proteins (HSPs), (G) antioxidant enzymes and (H) markers of mitochondrial stress.

Mitochondrial inefficiency was related to 41 proteins involved in the production and regulation of ATP. (a) Glycolysis: mitochondrial inefficiency was positively associated with three glycolysis enzymes (Fig. 6A). (b) β-oxidation: nestlings with higher mitochondrial inefficiency exhibited lower concentrations of proteins related to β-oxidation (Fig. 6B). β-oxidation is the metabolic process by which fatty acids are broken down in the mitochondria to generate acetyl-CoA, which enters the TCA cycle to produce energy. (c) TCA cycle: nestlings with higher mitochondrial inefficiency exhibited lower concentrations of proteins associated with the TCA cycle (Fig. 6C). The TCA cycle is a crucial metabolic pathway in cells that generates energy by oxidizing acetyl-CoA into carbon dioxide. It occurs in the mitochondrial matrix and is fundamental for producing ATP. (d) Oxidative phosphorylation: mitochondrial inefficiency was negatively associated with proteins involved in the process of mitochondrial oxidative phosphorylation complexes I, III, IV and V. Other proteins not linked to a specific complex but with functions in the ETS, such as CHCHD2 (coiled-coil-helix-coiled-coil-helix domain-containing protein 2) and MTFR1L (mitochondrial fission regulator 1 like), were also negative related to mitochondrial inefficiency (Fig. 6D). (d) Low-ATP signaling: we found a negative expression of two subunits of AMP kinase (AMPK) (Fig. 6E), which is upregulated when the cellular ATP pool is low.

#### Cellular stress

Mitochondrial inefficiency was positively related to: (a) heat shock proteins (Fig. 6F); (b) antioxidant enzymes that are upregulated to protect the cells from ongoing oxidative insult (Fig. 6G); and (c) proteins indicating mitochondrial stress (Fig. 6H).

### Relationship between body temperature and nest temperature observed in 2022

**Fig. 7.**
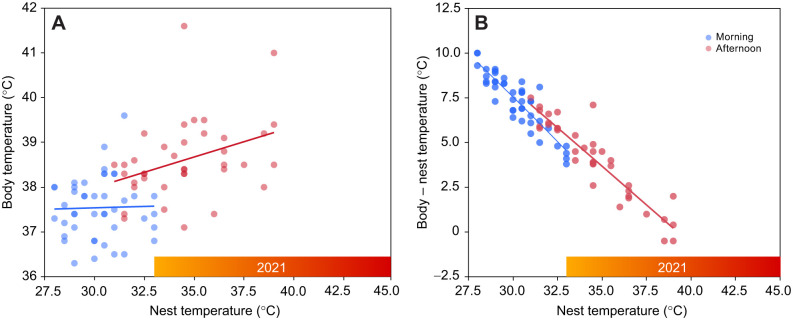
**Relationship between body temperature and nest temperature across different times of day.** (A) Relationship between body temperature and nest temperature recorded in the morning (blue) and in the afternoon (red) in 2022 (without heat wave). (B) Arithmetical difference between body and nest temperature in relation to the nest temperature recorded in the morning (blue) and in the afternoon (red) in 2022. The red bar on the *x*-axis of both graphs indicates the afternoon nest temperature recorded in 2021 during the heat wave. Dots represent individual data points, while the line indicates the fitted regression curve.

The average body temperature recorded in the morning was lower than the body temperature recorded in the afternoon (*F*_1,42.05_=56.92, *P*<0.0001; morning: 37.54±0.10°C, range 36.3–39.6°C, *n*=44: afternoon: 38.61±0.89°C, range 37.1–41.6°C, *n*=38; Fig. 7A). Body temperature was positively associated with nest temperature in the afternoon (β=0.137±0.06, *P*=0.02) but not in the morning (β=0.01±0.07, *P*=0.86; Fig. 7A). The difference between body and nest temperature (Δ*T*: body temperature–nest temperature) was smaller in the afternoon (*F*_1,36.58_=54.17, *P*<0.0001). This was shown also by the highly negative association between Δ*T* and nest temperature (*F*_1,59.44_=809.83, *P*<0.0001; Fig. 7B). Nest temperatures recorded in the afternoon were significantly higher in 2021 (average: 37.94±0.48°C, range: 33.0–44.5°C) than in 2022 (average: 34.74±0.38°C, range: 31.0–40.0°C) (*F*_1,36.58_=54.17, *P*<0.0001). The same trend was observed for the morning temperature, although the difference was not significant (*F*_1,38.94_=1.76, *P*=0.19, β=0.19±0.14).

## DISCUSSION

We showed that chicks living in warmer nests had higher mitochondrial inefficiency in coupling aerobic metabolism with ATP, which was associated with smaller body size at fledging. The proteome analysis revealed that this mitochondrial state was also associated with a downregulation of growth promoters and an increase of growth inhibitors, all determinants of organismal body size. The high nest temperatures related to this metabolic inefficiency persisted for several weeks in the study year, overlapping most of the steepest growth of nestlings (Fig. 1). This would explain why the physiological adjustments observed on the sampling day can represent what developing individuals experience over a longer period.

Shearwater chicks that grew up in warmer nests had mitochondria that were less efficient in coupling respiration with ATP production (higher LEAK and lower OXPHOS, whereas CMR and ETS remained invariant). Studies on fast-developing zebra finches that possess a thermoregulatory physiology that is well tuned to cope with high temperatures (Ton et al., 2021; Udino et al., 2021) found an increase in proton leak in nestlings exposed to high nest temperature (see Introduction), but CMR increased in parallel with proton leak, compensating for mitochondrial inefficiency and resulting in a similar size of chicks at fledging. Unlike what was observed in zebra finches, we found that CMR remained constant while LEAK increased and OXPHOS decreased, indicating that shearwaters have different strategies to respond to higher temperatures. The bioenergetic evidence was supported at the biochemical level, where nestlings with higher mitochondrial inefficiency showed a lower expression of several enzymes involved in OXPHOS and, more specifically, complex I and ATP synthase (Fig. 5A–D). The proteomic signature of a downregulation of key enzymes involved in the oxidative phosphorylation machinery suggests that this was a regulated process rather than a side effect caused by higher body temperature altering the permeability of cellular membranes (see below for further discussion on this point).

In a subsequent examination conducted the following year (i.e. when no heat waves occurred), we showed that the average increase in body temperature observed during the hottest part of the day, compared with the cooler morning conditions, was only 1.08± 0.15°C, thus compatible with chicks being capable of thermoregulation; however, the range was quite wide (range 0–4°C; Fig. 7). The observed patterns in body temperature suggest that nestlings exhibit a degree of thermoregulatory capability under cooler conditions, but this capacity appears to weaken as environmental temperatures rise. In the morning, when nest temperatures were lower, nestlings were able to maintain body temperatures relatively independently of their surroundings. However, during hotter afternoon conditions, body temperatures became more closely linked to nest temperatures, suggesting a diminished ability to buffer against thermal challenges. This connection, particularly when nest temperatures approached or exceeded 40°C, indicates that nestlings may have been nearing their thermoregulatory limits. Given that nest temperatures in 2021 reached as high as 44.5°C, it is plausible that nestlings were experiencing significant thermal stress, as several proteomic parameters would suggest (addressed below). We did not observe any increase in metabolic rate (CMR, which serves as a proxy for organismal metabolic rate; Casagrande et al., 2023) with rising temperatures (however, CMR was a meaningful variable as it was positively associated with body mass) (see Table S3a), suggesting that chicks did not expend energy to cope with high temperature. Further investigations are needed to determine whether this was a strategy to favor passive body heat dispersal (hyperthermia), which may help reduce the energetic and water costs of active thermoregulation (McKechnie and Wolf, 2010) or it was a consequence of an immature thermoregulatory system.

We found a higher concentration of three key enzymes of glycolysis (e.g. pyruvate kinase M1/M2; Fig. 6C) and a lower concentration of five β-oxidation enzymes (Fig. 6B) in nestlings with higher temperature-associated mitochondrial inefficiency (Fig. 6C). Although glycolysis provides quick energy, it is less efficient in terms of ATP yield per glucose molecule compared with complete oxidation through oxidative phosphorylation. A further support to the glycolysis shift comes from the upregulation of the growth factor FGFR1 (see Table S3 for further details), which triggers the metabolic change from oxidative phosphorylation to glycolysis. Overall, these results suggest that cells compensated for the mitochondrial uncoupling state by upregulating glycolysis, which is able to deplete glucose levels, another route by which cell growth could have been constrained. The observed decrease in β-oxidation and increase in glycolysis-related proteins suggests a metabolic shift toward carbohydrate metabolism, even in chicks consuming a fish-oil-rich diet. Fish oil consists primarily of triglycerides and free fatty acids, with negligible carbohydrate content. However, the glycerol backbone of triglycerides can be converted into glucose through gluconeogenesis, a pathway particularly active in birds (McCue, 2010). Whereas fatty acids from fish oil are typically oxidized via β-oxidation to produce acetyl-CoA for ATP production, the conversion of acetyl-CoA back into glucose is not possible because of metabolic constraints (Weber and Haman, 2004). Instead, under heat stress, metabolic regulation often suppresses lipid oxidation to minimize excess heat production, favoring carbohydrate metabolism, which is more oxygen efficient and generates less heat per unit of ATP produced (McKechnie and Wolf, 2019). This shift highlights how metabolic flexibility can allow chicks to prioritize carbohydrates for energy, despite a high dietary lipid intake, to adapt to thermal stress. Additionally, it is worth noting that ATP production via glycolysis generates less heat than aerobic metabolism in the mitochondria. Thus, the observed shift towards glycolysis in this scenario may represent a deliberate strategy to mitigate heat production when producing ATP. Actually, AMPK, a kinase that increases when ATP level is low (here represented by PRKAA1 and PRKAA2; Fig. 5E), decreased when mitochondrial inefficiency was higher. AMPK can affect cell growth by, for example, inhibiting mTOR (Hardie et al., 2012). Although puzzling, this finding could indicate that in conditions of prolonged mitochondrial inefficiency, AMPK might be downregulated to prevent any additional perturbation of cell growth. Alternatively, the decrease in AMPK could suggest that the glycolysis pathway compensates for the reduced mitochondrial ATP production, providing sufficient energy to prevent the activation of AMPK under these conditions. This hypothesis aligns with the idea that glycolysis can serve as a rapid, alternative source of ATP.

The downregulation of several enzymes of the Krebs cycle (or TCA cycle) suggests that high temperatures could have compromised not only ATP synthesis but also the anabolic functions of mitochondrial metabolism, further contributing to impaired growth performance. The observed downregulation of several subunits of complex I could result in reduced NADH oxidation, leading to a redox imbalance characterized by an elevated NADH/NAD^+^ ratio. This imbalance is likely to inhibit key enzymes of the Krebs cycle (Camus et al., 2023; Lane, 2020), thereby limiting the production of critical precursors that are essential for the biosynthesis of amino acids, nucleotides and lipids, indispensable for cell growth and proliferation (Harrison and Lane, 2018). Furthermore, although glycolysis may partially compensate for reduced mitochondrial ATP production, it cannot sustain the supply of these carbon skeleton precursors, and this amplifies the negative impact of exposure to high temperature on cellular growth.

### Mitochondrial inefficiency correlates negatively with growth factors and downstream growth promoters

The reduction in body size was also supported at the proteomic level, where mitochondrial inefficiency was negatively associated with growth promoters and positively associated with growth suppressors (Fig. 5A–C). Specifically, mitochondrial inefficiency observed in warmer nests was negatively associated with insulin-like growth factor 2 binding protein (IGF2BP; Fig. 5A), involved in cell proliferation, differentiation and survival, influencing tissues such as muscles and bones (Allard and Duan, 2018). IGF2BP also influences signaling pathways involving several functional receptors of the growth axis (Firth and Baxter, 2002). Mitochondrial inefficiency was positively related to insulin-like growth factor binding protein 3 (IGFBP3; Fig. 5A), which is the predominant protein in the bloodstream responsible for binding IGF-1, thereby modulating its growth-promoting effects (Yamada and Lee, 2009). Beyond its relationship with IGF-1, the primary physiological function of IGFBP3 is to suppress cell proliferation and growth (Yamada and Lee, 2009), a function that is exploited when used as a tumor-suppressive agent (Kwon et al., 2023). Nestlings exhibiting warm-temperature-related heightened mitochondrial inefficiency had diminished levels of transforming growth factor beta1 (TGFB1), which modulates the growth and differentiation of diverse cell types, and of fibroblast growth factor receptor 1 (FGFR1; Fig. 5A), a receptor that binds to fibroblast growth factors, a broad family of multifunctional signaling proteins endowed with metabolic adjustments under cellular stress. For example, FGFR1 orchestrates intracellular responses to metabolic stress upon activation by specific fibroblast growth factors, such as fibroblast growth factor 21 (FGF21), a blood biomarker of inefficient oxidative phosphorylation (Hubens et al., 2022). Importantly, FGFR1 plays a significant role in reprogramming cellular metabolism, promoting a transition from oxidative phosphorylation to glycolysis. This function aligns with the present study's findings, which showed an increase in enzymes responsible for glycolysis (Fig. 6D). Taken together, these findings bridge inefficient cell energy metabolism and organismal growth constraint.

### Lower levels of downstream growth promoters in nestlings showing temperature-associated mitochondrial inefficiency

We found that the mitochondrial inefficiency in coupling respiration with ATP production, associated with smaller nestling sizes, was linked with changes in proteins interacting with mTOR (Fig. 5B). Among the upstream activators are serine/threonine kinase (STK11), which exhibited a significant negative association with mitochondrial inefficiency, and late endosomal/lysosomal adaptor (LMATOR2), which was positively associated with mitochondrial inefficiency (Fig. 5B). STK11 is a known inhibitor of the mTOR signaling pathway via AMPK (indeed, we also found a negative expression of two subunits of this complex kinase: PRKAA1 and PRKAA2; Fig. 5B) (Hardie et al., 2012). Similarly, LAMTOR2 activates mTOR when sensing high levels of amino acids (Bar-Peled et al., 2012). A possible interpretation is that nestlings with diminished levels of mTOR inhibitor (i.e. Stk11) and enhanced levels of mTOR activator (LMATOR2; Fig. 5B) might have downregulated energy-sensing mechanisms related to reduced ATP production. However, this interpretation needs further investigation, especially in light of what was found for the upstream mTOR inhibitor, tuberous sclerosis complex 2 (TSC2), which was positively associated with mitochondrial inefficiency (Fig. 5B). It has been shown that TSC2 acts as a suppressor of mTOR activity that leads to reduced protein synthesis and growth (Inoki et al., 2002). This is consistent with the findings of the present study as well as the negative association with mitochondrial inefficiency of activators of mTOR – regulatory-associated protein of mTOR (RPTOR) and RAC-beta serine/threonine-protein kinase (AKT2; Fig. 6B). RPTOR is an essential component of mTOR that helps recruit substrates to mTOR and is crucial for the activation and function of the complex, thereby regulating cellular growth, metabolism and survival. AKT2, a pivotal component of the insulin signaling pathway and an integral component of the mTOR signaling cascade, acts upstream to activate mTOR. By modulating the balance between anabolism and catabolism, AKT2 essentially orchestrates the direction of metabolic pathways to support cell growth and proliferation. For instance, upon activation, AKT2 promotes protein synthesis, lipid biosynthesis and cell cycle progression while concurrently inhibiting pro-apoptotic factors. Thus, a negative association between mitochondrial inefficiency and AKT2 suggests that disruptions in the optimal functioning of AKT2 can potentially impair cellular biosynthetic processes, thereby influencing growth outcomes in the context of warm-associated mitochondrial challenges. mTOR is a complex kinase made by the integration of different units that can act independently from each other and that control specific pathway related to growth; thus, it is not surprising that contrasting signals were acting at the upstream level (Dibble and Manning, 2013). The outcome of the mTOR state was clearer when referring to downstream growth promoters (CREB1, cyclic AMP-responsive element-binding protein; GICYF2, GRB10-interacting GYF protein 2; NR2C2, nuclear receptor subfamily 2 group C member 2; SRC, proto-oncogene tyrosine-protein kinase SRC; STRAP, serine/threonine kinase receptor-associated protein; TYK2, non-receptor tyrosine-protein kinase TYK2; SMAD2, mothers against decapentaplegic homolog 2; BMP2K, BMP2 inducible kinase; LNPK, endoplasmic reticulum junction formation protein lunapark; Fig. 5C), which were all negatively associated with high-temperature-associated mitochondrial inefficiency; in turn, this might explain the effect of warm temperature on growth reduction in warmer nests.

### Mitochondrial inefficiency was related to heat-shock proteins, antioxidant enzymes and mitochondrial stress response

A significant relationship also emerged between mitochondrial inefficiency and markers of cellular stress or damage prevention. Specifically, there was a positive association with heat shock proteins (HSPs), which serve as critical cellular guardians, especially during stressful conditions, by assisting in protein folding, preventing protein aggregation and facilitating the degradation of damaged proteins. Although their primary role lies in ensuring cellular proteostasis, studies suggest potential trade-offs associated with elevated HSP levels, which may divert energy and resources away from regular cellular processes including growth (Budzynski and Sistonen, 2017; Pirkkala et al., 2001). Although HSPs are often induced by heat stress, their elevation in our study may reflect a direct effect not only of external temperature, but also of cell stress, which was observed in the present study (developed below). Furthermore, HSPs have been implicated in the modulation of programmed cell death pathways (Beere, 2005), emphasizing that the protective role of HSPs may come at the cost of growth inhibition and a consequent achievement of smaller bodies (Chen et al., 2018; Du and Shine, 2015).

On the same line is the finding that mitochondrial inefficiency was positively related to mitochondrial stress markers and antioxidant enzymes that act to neutralize the impacts of oxidative stress (Fig. 6F). The upregulation of enzymes such as glutamate-cysteine ligase catalytic subunit (GCLC; essential for glutathione synthesis), glutathione peroxidase 1 (GPX1), glutathione S-transferase alpha-1-2 (GSTA1-2) and peroxiredoxin-6 (PRDX6) indicates that the cell was responding to offset mitochondrial disturbances arising because of high-temperature-associated mitochondrial inefficiencies. Oxidative stress per se can impede growth because of direct cell damage (Lee et al., 2011; Smith et al., 2016) or because endogenous antioxidants are costly to produce and must be traded off with growth. Indeed, it has been shown that glutathione and GPX upregulations can impact survival in the long term (Casagrande and Hau, 2018; Romero-Haro et al., 2023). Overall, these findings suggest that thermal stress in warmer nests triggered cellular and mitochondrial stress responses, which may have significant implications for growth and long-term survival.

### Further considerations

One weakness of the study is that we considered the relationship between mitochondrial traits measured during the peak of the growth curve with the final body size. It is possible that mitochondrial traits observed at the peak growth phase may differ from those manifested at the cessation of growth, where energy requirements and allocation strategies might shift. Although our study does not capture this longitudinal aspect, it lays the groundwork for subsequent research aimed at discerning whether mitochondrial characteristics measured during rapid growth persist into later life stages or are subject to dynamism. We are not aware of longitudinal studies carried out in birds that considered the persistency of mitochondrial phenotype in relation to growth. However, we believe that even if mitochondrial traits change in later phases of growth or life, the association we observed remains biologically meaningful because the measurement represents a critical phase of shearwater growth.

### Conclusions

Body size can drive performance, determine fitness and have population- and community-level repercussions (Pettersen et al., 2019; Sauve et al., 2021). Consequently, it represents an important means through which climate warming could reshape ecosystems and populations (Hallam and Harris, 2023; Louthan et al., 2021; Pontzer, 2021). Knowing the physiological mechanisms that regulates this phenotypic plasticity can significantly improve our ability to predict the actual impacts of increased temperatures. For instance, the presence of ample nutrients might mitigate the effects of warm temperatures, provided that pathways such as mTOR are not inhibited owing to the absence of ‘high energy’ signals.

It is unlikely that the observed physiological responses to nest conditions increased resilience to heat while individuals were in the nest. Although the size shifts align with Bergmann's rule, they are probably too small to make a meaningful contribution (Nord et al., 2024). Additionally, a reduction in beak size contradicts Allen's rule, which predicts that larger appendages would favor heat dissipation (McKechnie and Wolf, 2019). Furthermore, smaller sizes may not offer advantages during adulthood, especially when individuals face significantly cooler environmental conditions (Choy et al., 2021). Future studies should explore how these size-related traits influence adult survival and fitness across different environmental contexts. Notably, markers of cell stress were found, indicating that resources are diverted from growth to cope with thermal conditions. These physiological alterations are responses that free-living birds, and likely other endotherms, must trigger to cope with a warming world, and we think they deserve further investigation.

## Supplementary Material

10.1242/jexbio.249880_sup1 Supplementary information
