# Supplementary material for: Linking warmer nest temperatures to reduced body size in seabird nestlings: possible mitochondrial bioenergetic and proteomic mechanisms
Source: J Exp Biol. 2025 Mar 28;228(6):jeb249880. doi: 10.1242/jeb.249880 (PMC12243454; doi:10.1242/jeb.249880)
Supplement: Supplementary information [file jexbio-228-249880-s1.pdf]

Journal of Experimental Biology • Supplementary information

SUPPLEMENTARY MATERIALS AND METHODS

The role of glucocorticoids in mediating environmental temperature effect on growth

Another physiological mechanism that could influence the relationship between temperature, metabolism and growth plasticity is the upregulation of glucocorticoids, which play a fundamental role in keeping homeostasis in stressful conditions (Mentesana and Hau, 2022), promoting metabolic changes (Casagrande et al., 2020; Dupont et al., 2019; Picard et al., 2014) and mediating trade-offs between metabolism and growth (Braun et al., 2013; Casagrande et al., 2020). Glucocorticoids exert a fundamental function in supporting metabolism: they are expected to decrease with increasing temperature, below the TNZ (i.e., moving from low temperatures to thermoneutrality; (Mentesana and Hau, 2022)), to revert this relationship in the upper thermal limit). To date, we are not aware of any study that considered the role of these hormones in cell metabolism related to thermoregulation at high temperatures and growth.

We predicted that individuals exposed to higher nest temperature have higher corticosterone concentrations, which may additionally mediate lower growth rates while enhancing metabolic rate and mitochondrial inefficiency (Casagrande et al., 2020; Tilgar, 2019).

Mitochondrial bioenergetics

Oxygen consumed by aerobic metabolism during mitochondrial respiration was measured in red blood cells (RBCs, (Casagrande et al., 2020; Stier et al., 2017) with a Clark electrode high resolution respirometer chamber (Oxygraph-2k, Oroboros Instruments, Innsbruck, Austria). The full protocol has been described in the supplemental material. Blood samples were centrifuged at 2000 x g for 10 min, and plasma was separated from blood cells for corticosterone analysis. RBCs (ranging between 25 and 80 µL) were transferred into 1 ml of cold Mir05 buffer for washing (0.5 mmol l−1 EGTA, 3 mmol l−1 MgCl2, 60 mmol l−1 potassium lactobionate, 20 mmol l−1 taurine, 10 mmol l−1 KH2PO4, 20 mmol l−1 Hepes, 110 mmol l−1 sucrose, 15 mmol l−1 fatty acid-free bovine serum albumin, pH 7.1). To limit contamination of RBCs with leukocytes, the sample was taken from the bottom of the tube. RBCs were then centrifuged at 500 x g for 5 min and after supernatant discard, resuspended in 1 ml of MiR05 buffer already equilibrated at 40°C in a Clark electrode high resolution respirometer chamber (Oxygraph-2k, Oroboros Instruments, Innsbruck, Austria).

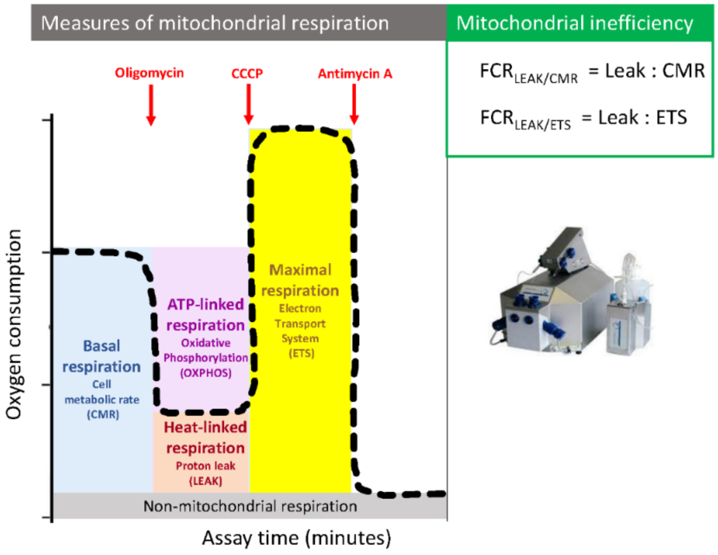

Fig. S1. Description of the protocol used to measure mitochondrial traits using high-resolution respirometry; further details on the protocol are provided in the main text. From these primary measurements, derivative traits were computed for statistical analysis.

Correlations among mitochondrial traits

Pearson's r

	cmr	oxphos	leak	ets	fcr1	fcr2
cmr	1.0000	0.8504	0.3150	0.8649	-0.5057	-0.3944
oxphos	0.8504	1.0000	-0.2314	0.6654	-0.8611	-0.7596
leak	0.3150	-0.2314	1.0000	0.3990	0.6183	0.6411
ets	0.8649	0.6654	0.3990	1.0000	-0.3174	-0.3911
fcr1	-0.5057	-0.8611	0.6183	-0.3174	1.0000	0.9170
fcr2	-0.3944	-0.7596	0.6411	-0.3911	0.9170	1.0000

P- values

	cmr	oxphos	leak	ets	fcr1	fcr2
cmr	<.0001	<.0001	0.0310	<.0001	0.0003	0.0061
oxphos	<.0001	<.0001	0.1176	<.0001	<.0001	<.0001
leak	0.0310	0.1176	<.0001	0.0055	<.0001	<.0001
ets	<.0001	<.0001	0.0055	<.0001	0.0297	0.0066
fcr1	0.0003	<.0001	<.0001	0.0297	<.0001	<.0001
fcr2	0.0061	<.0001	<.0001	0.0066	<.0001	<.0001

Journal of Experimental Biology • Supplementary information

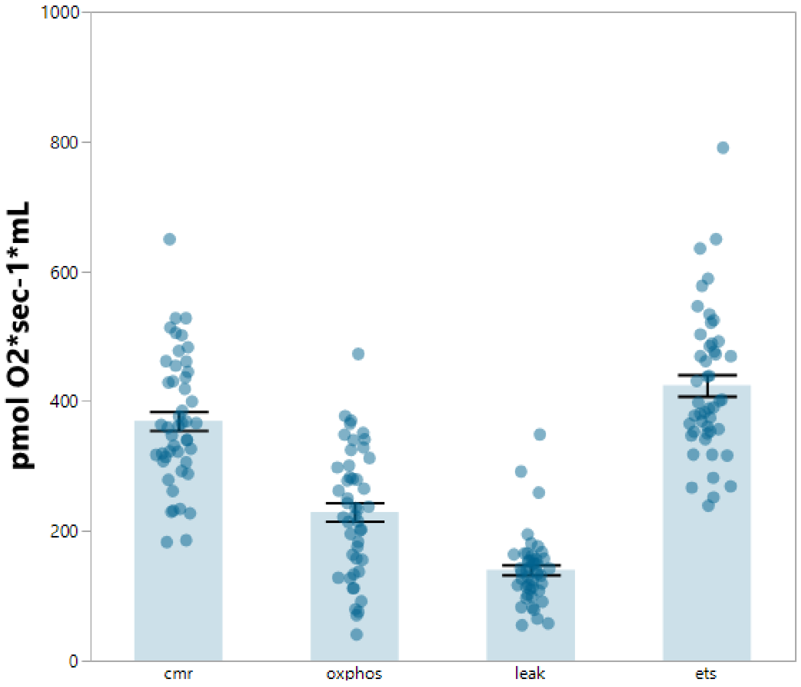

Fig. S2. Mitochondrial bioenergetic traits measured in the 47 nestlings of the study.

Proteome analysis

The untargeted proteome was analyzed through liquid chromatography mass spectrometry (LC-MS) by the Max Planck Institute of Biochemistry. Blood cells in NBS buffer were centrifuged at 1500 x g. The supernatant was discarded and the pellet was resuspended in 400 µL of SDC buffer (1% sodium deoxycholate, 40 mM 2-chloroacetamide -Sigma-Aldrich-, 10 mM tris(2-carboxyethyl) phosphine -TCEP; PierceTM, Thermo Fisher Scientific- in 100 mM Tris, pH 8.0) and incubated at 95 °C for 5 min followed by sonification in a Bioruptor plus system (Diogenode) for 10 x 30 s at high intensity. Heating and sonification was repeated once more. Afterwards, the samples were diluted with 400 µL of water (LC-MS grade) and supplemented with 1 µg of LysC followed by incubation at 37 °C for 2 hours. After addition of 2 µg of trypsin (Promega), the samples were digested overnight at 37°C. The peptides were then acidified with trifluoroacetic acid (final concentration of 1%). The samples were centrifuged to remove the precipitated SDC. The supernatant was diluted 1:10 and

Journal of Experimental Biology • Supplementary information

3 µL of the samples were loaded on Evotips (Evotip pure, Evosep) following the standard clean-up protocol from Evosep.

LC-MS/MS data acquisition. Evotips were eluted onto a 15-cm column (PepSep C18 15 cm x 15 cm, 1.5 µm, Bruker Daltonics) via the Evosep One HPLC system. The column was heated to 50 °C and peptides were separated using the 30 SPD method. Using the nanoelectrospray interface, eluting peptides were directly sprayed onto the timsTOF Pro mass spectrometer (Bruker Daltonics). Data acquisition on the timsTOF Pro was performed using timsControl. The mass spectrometer was operated in data-independent (DIA) PASEF mode. Analysis was performed in a mass scan range from 100-1700 m/z and an ion mobility range from 1/K0 = 0.70 Vs cm-2 to 1.30 Vs cm-2 using equal ion accumulation and ramp time in the dual TIMS analyzer of 100 ms each at a spectra rate of 9.52 Hz. Dia-PASEF scans were acquired at mass scan range from 350.2-1199.9 Da and an ion mobility range from 1/K0 = 0.70 Vs cm-2 to 1.30 Vs cm-2. Collision energy was ramped linearly as a function of the mobility from 45 eV at 1/K0 = 1.30 Vs cm−2 to 27 eV at 1/K0 = 0.85 Vs cm−2. In complete, 42 diaPASEF windows were distributed to one TIMS scan each at switching Th precursor isolation windows (see file “diaParameters.txt”) which led to an estimated cycle time of 2.21 seconds. The ion mobility dimension was calibrated linearly using three ions from the Agilent ESI LC/MS tuning mix (m/z, 1/K0: 622.0289, 0.9848 Vs cm-2; 922.0097, 1.1895 Vs cm-2; 1221.9906, 1.3820 Vs cm2).

Data Analysis – Raw data were processed using the Spectronaut 17.0 in directDIA+ (library-free) mode. Shortly, the peak list was searched against a predicted library of the Calonectris borealis (Cory's shearwater) database from uniport (14417 entries, downloaded in 2022). Cysteine carbamidomethylation was set as static modification, and methionine oxidation and N-terminal acetylation as variable modifications. The match-between-run option was enabled, and proteins were quantified across samples using the label-free quantification (MaxLFQ) at the MS2 level.

Molecular sex determination

Nestling sex was determined from blood using a molecular protocol validated for the model species (Tagliavia et al., 2023). Briefly, DNA was extracted using a drop of blood dried onto filter paper using 50 µl of lysis solution (200 mM KOH, 20 mM Na2EDTA, 0.25% Triton X-100), after

Journal of Experimental Biology • Supplementary information

incubation at ambient temperature with the addition of six volumes of a neutralization solution (50 mM Tris-HCl, 0.0025% Bromothymol Blue, BTB). Two sets of primers - ProcZ and ProcW - specifically targeting conserved regions of CHD-Z and CHD-W of Procellariforms were used. The ready-to-use reaction mix consisted of 1X enzyme buffer, dNTP 1.4 mM, MgSO4 8mM (final total concentration), 1.6 µM each FIP/BIP, 0.2 µM each F3/B3, 0.4 µM each Loop F/B (in CdZ mix only), eight units of Bst 2 Warm-Start DNA polymerase (New England Biolabs), 2% sucrose, 700 µM Eriochrome Black T (EBT), 5% DMSO. The mix was incubated for 20-60 min at 55°C and products were analyzed by electrophoresis on 3% agarose gel.

Corticosterone assay

Plasma corticosterone concentrations were measured in duplicate with a corticosterone enzyme immunoassay following the protocol provided by the manufacturer (Cat. No. K014-H1; Corticosterone ELISA Kit, Arbor Assays). Plasma corticosterone concentrations were measured in duplicate following a double diethyl ether extraction of a 15 µL plasma sample. Samples were reconstituted in assay buffer provided by the manufacturer and allowed to reconstitute over-night at 4°C before analysis. Two control samples (stripped chicken plasma with 10 and 5 ng mL-1 added corticosterone) were taken through the same extraction and analysis procedures as the plasma samples. They were used also to calculate inter-plate coefficient of variation (CV) that was 4.21.40 ± 0.63 %, and the intra-plate CV was, that was 2.42 ± 0.45 %.

We predicted that individuals exposed to higher nest temperature have higher corticosterone concentrations, which may additionally mediate lower growth rates while enhancing metabolic rate and mitochondrial inefficiency (Casagrande et al., 2020; Tilgar, 2019). To test whether nest temperature was related to circulating level of corticosterone (question 3) we ran a model with corticosterone concentration as response variable and nest temperature at sampling as predictor, while correcting for the duration of sampling, as corticosterone concentrations increase immediately after capture (Casagrande and Hau, 2018).

The observed metabolic responses in warmer nests were found to be not related with circulating corticosterone levels, (nest temperature: β=-0.031±0.08, $t_{(1,45)}$=-0.37, p=0.71), sex ((male): β=-

Journal of Experimental Biology • Supplementary information

0.08±0.25, $t_{(1,45)}$=-0.32, p=0.75); or sampling duration ($t_{(1,45)}$=0.78, p=0.44; effect size: f^2=0.02). Corticosterone was generally low. Previous research has indicated that high levels of circulating corticosterone are predicted—and indeed observed—in birds subjected to thermal stress (Cooper et al., 2020; Mentesana and Hau, 2022; Rubalcaba and Jimeno, 2022). Yet, some studies have suggested that the endocrine stress response could potentially be suppressed in high-temperature environments, indicating that corticosterone may not represent a reliable biomarker for thermal stress (Schradin et al., 2023). Such an adjustment might be beneficial for the physiological conditions of nestlings to avoid long-term negative consequences that an upregulation of the HPA axis might have (Hau et al., 2016).

Table S1. Models explaining body size at fledging using measures of nest temperatures.

	**Beak size**		**Body mass**
**Nest temperature °C**	**R^2 - AIC**	**Nest temperature °C**	**R^2 - AIC**
**Morning T+ Sex**	**0.57 - 118.66**	**Morning T+ Sex**	**0.27 - 523.97**
**HW DT Average T+ Sex**	**0.52 - 125.60**	**HW DT Max T+ Sex**	**0.22 - 531.35**
**Whole cycle Max T+ Sex**	0.49 - 126.50	**Whole cycle Max T+ Sex**	0.18 - 532.53
**HW DT Max T+ Sex**	0.48 - 127.42	**HW DT Average T+ Sex**	0.19 - 532.98
**Whole cycle Average T+ Sex**	0.48 - 127.77	**Whole cycle Average T+ Sex**	0.15 - 534.21
**HW DT Min T+ Sex**	0.46 - 129.09	**HW DT Min T+ Sex**	0.14 - 534.76
**Whole cycle Min T+ Sex**	0.46 - 129.41	**Whole cycle Min T+ Sex**	0.14 - 536.24

Notes. “Sex” of chicks was always included, n=47 for all the models. HW=Heat Wave from 20/07/24 to 20/08/24. DT=Day Time from 8:00 to 20:00 h. In bold, models that reached statistical significance.

Table S2. Effects of temperature-mediated mitochondrial traits on body size traits at fledging (question 4).

Variable (R2-f^2)	Estimate (±s.e.)	t $_{(1,44)}$	p
a) Beak size-PC1 (0.47 – 0.98)			
CMR	0.001 (±0.001)	0.88	0.38
**Sex(male)**	**1.72(±0.28)**	**6.19**	**<0.0001**
b)Beak size-PC1 (0.51 – 1.04)			
**OXPHOS**	**0.003(±0.001)**	**2.23**	**0.0308**
**Sex(male)**	**1.75(±0.26)**	**6.63**	**<0.0001**
b)Beak size-PC1 (0.52 – 1.32)			
**LEAK**	**-0.005(±0.002)**	**-2.33**	**0.0245**
**Sex(male)**	**1.64(±0.26)**	**6.27**	**<0.0001**
c)Beak size-PC1 (0.54 – 1.73)			
$FCR_{LEAK/CMR}$	**-2.27(±0.78)**	**-2.89**	**0.006**
**Sex(male)**	**1.76(±0.25)**	**6.89**	**<0.0001**
**Variable (R2-f^2)**	**Estimate (±s.e.)**	**t $_{(1,44)}$**	**p**
a)Body mass (0.21 - 0.27)			
**CMR**	**0.20 (±0.10)**	**2.06**	**0.045**
**Sex(male)**	**62.27(±19.93)**	**3.12**	**0.003**
b)Body mass (0.25 - 0.34)			
**OXPHOS**	**0.24(±0.10)**	**2.51**	**0.016**
**Sex(male)**	**61.81(±19.43)**	**3.18**	**0.003**
c)Body mass (0.15 - 0.18)			
LEAK	-0.11(±0.18)	-0.60	0.55
**Sex(male)**	**54.99(±20.58)**	**2.67**	**0.01**
d)Body mass (0.22 - 0.28)			
**$FCR_{LEAK/CMR}$**	**-127.62(±60.69)**	**-2.10**	**0.04**
**Sex(male)**	**59.99(±19.75)**	**3.04**	**0.004**

Note: values in brackets after the dependent variable name report R2 and Cohen's effect size.

Table S3. Full names of proteins depicted in figure 4 and 5.

Figure 4 panel	System	Protein	ProtName
A	growth/signalling/growth_factors inhibitor	**Igf2bp3**	**Insulin Like Growth Factor 2 Binding Protein 3**
A	growth/signalling/growth factors	**Igfbp3**	**Insulin Like Growth Factor Binding Protein 3**
A	growth/signalling/growth_factors inhibitors	**Tgfb1**	**Transforming growth factor beta-1 proprotein**
A	growth/signalling/growth_factors receptor	**Fgfr1**	**Fibroblast growth factor receptor 1**
B	growth/signalling/TOR/upstream/inhibitor	**Stk11**	**Serine/Threonine Kinase 4**
B	growth/signalling/TOR/upstream/activator	**Lamtor2**	**Late Endosomal/Lysosomal Adaptor, MAPK And MTOR activator**
B	growth/signalling/TOR/upstream/inhibitor	**Tsc2**	**Tuberous sclerosis complex 2**
B	growth/signalling/TOR/activator	**Rptor**	**Regulatory-associated protein of mTOR**
B	growth/signalling/growth promoter/TOR/upstream/protein biosynthesis	**Akt2**	**RAC-beta serine/threonine-protein kinase**
C	growth/signalling/growth promoter	**Creb1_1**	**Cyclic AMP-responsive element-binding protein 1**
C	growth/signalling/growth promoter	**Gigyf2**	**GRB10-interacting GYF protein 2**
C	growth/signalling/growth promoter	**Nr2c2**	**Nuclear Receptor Subfamily 2 Group C Member 2**
C	growth/signalling/growth promoter	**Src**	**Proto-oncogene tyrosine-protein kinase Src**
C	growth/signalling/growth promoter	**Strap**	**Serine/threonine kinase receptor-associated protein**
C	growth/signalling/growth promoter	**Tyk2**	**Non-receptor tyrosine-protein kinase TYK2**
C	growth/signalling/growth promoter	**Smad2**	**Mothers against decapentaplegic homolog 2**
C	growth/signalling/growth promoter	**Bmp2k**	**BMP2 Inducible Kinase**
C	growth/signalling/growth promoter	**Lnpk**	**Endoplasmic reticulum junction formation protein lunapark**
**Figure 5 panel**	**System**	**Protein**	**ProtName**
A	ATP/gycolysis	**Eno2**	**Enolase 2**
A	ATP/gycolysis	**Aldob**	**Fructose-bisphosphate aldolase B**
A	ATP/glycolysis	**Pkm**	**Pyruvate kinase M1/M2**
B	mito/ATP/beta-oxidation	**Acaa2**	**3-ketoacyl-CoA thiolase**
B	mito/ATP/beta-oxidation	**Cpt2**	**Carnitine palmitoyltransferase 2**
B	mito/ATP/beta-oxidation	**Echs1**	**Enoyl-CoA Hydratase, Short Chain 1**
B	mito/ATP/beta-oxidation	**Hadhb**	**Trifunctional enzyme**

Journal of Experimental Biology • Supplementary information

			**subunit beta**
**B**	mito/ATP/beta-oxidation	**Hsd17b10**	**Hydroxysteroid 17-Beta Dehydrogenase 10**
**C**	mito/ATP/TCA cycle	**Idh2**	**Isocitrate Dehydrogenase (NADP(+)) 2**
**C**	mito/ATP/TCA cycle	**Idh3a**	**Isocitrate Dehydrogenase (NADP(+)) 3**
**C**	mito/ATP/TCA cycle	**Idh3b**	**Isocitrate dehydrogenase [NAD] subunit beta**
**C**	mito/ATP/TCA cycle	**Idh3g**	**Isocitrate Dehydrogenase (NADP(+)) g**
**C**	mito/ATP/TCA cycle	**Sdhb**	**Succinate Dehydrogenase Complex Iron Sulfur Subunit B**
**C**	mito/ATP/TCA cycle	**Suclg1**	**Succinate--CoA ligase [ADP/GDP-forming] subunit alpha**
**C**	mito/ATP/TCA cycle	**Suclg2**	**Succinate-CoA ligase [ADP-forming] subunit gamma**
**C**	mito/ATP/TCA/gycolysis linked	**Dlat**	
**D**	mito/ATP/oxidative phosphorylation/complex I	**ND3**	**NADH dehydrogenase subunit 3**
**D**	mito/ATP/oxidative phosphorylation/complex I	**ND5**	**NADH-ubiquinone oxidoreductase chain 5**
**D**	mito/ATP/oxidative phosphorylation/complex I	**Ndufa1**	**NADH ubiquinone oxidoreductase subunit**
**D**	mito/ATP/oxidative phosphorylation/complex I	**Ndufa12**	**NADH ubiquinone oxidoreductase subunit**
**D**	mito/ATP/oxidative phosphorylation/complex I	**Ndufa2**	**NADH ubiquinone oxidoreductase subunit**
**D**	mito/ATP/oxidative phosphorylation/complex I	**Ndufab1**	**NADH ubiquinone oxidoreductase subunit**
**D**	mito/ATP/oxidative phosphorylation/complex I	**Ndufaf4**	**NADH dehydrogenase [ubiquinone] 1 alpha subcomplex assembly factor 4**
**D**	mito/ATP/oxidative phosphorylation/complex I	**Ndufb3**	**NADH dehydrogenase [ubiquinone] 1 beta subcomplex subunit 3**
**D**	mito/ATP/oxidative phosphorylation/complex I	**Ndufb9**	**NADH:Ubiquinone Oxidoreductase Subunit B9**
**D**	mito/ATP/oxidative phosphorylation/complex I	**Ndufs8**	**NADH ubiquinone oxidoreductase subunit**
**D**	mito/ATP/oxidative phosphorylation/complex I	**Ndufv2**	**NADH dehydrogenase [ubiquinone] flavoprotein 2**
**D**	mito/ATP/oxidative phosphorylation/Complex I	**Tmem126a**	**Transmembrane protein 126A**
**D**	mito/ATP/oxidative phosphorylation/complex I	**Zmat3**	**Mitochondrial NADH-ubiquinone oxidoreductase assembly factor 3**
**D**	mito/ATP/oxidative phosphorylation/complex III	**Uqcr11**	**Cytochrome b-c1 complex subunit 10**
**D**	mito/ATP/oxidative phosphorylation/complex III	**Uqcrh**	**Cytochrome b-c1 complex subunit 6, mitochondrial**

Journal of Experimental Biology • Supplementary information

**D**	mito/ATP/oxidative phosphorylation/complex III	**Bloc1s2**	**Biogenesis of lysosome-related organelles complex 1 subunit 2**
**D**	mito/ATP/oxidative phosphorylation/complex III	**Cybc1**	**Cytochrome b-c1 complex subunit 1**
**D**	mito/ATP/oxidative phosphorylation/complex III	**Cyc1**	**Cytochrome c isoform 1**
**D**	mito/ATP/oxidative phosphorylation/complex III	**CYTB**	**Cytochrome b**
**D**	mito/ATP/oxidative phosphorylation/complex III	**Uqcrb**	**Ubiquinol-cytochrome c reductase binding protein**
**D**	mito/ATP/oxidative phosphorylation/between complex III and IV	**Cyc**	**Cytochrome C**
**D**	mito/ATP/oxidative phosphorylation/complex IV	**COX2**	**Cytochrome c oxidase subunit 2**
**D**	mito/ATP/oxidative phosphorylation/complex IV	**Cox6a1**	**Cytochrome C Oxidase Subunit 4I1**
**D**	mito/ATP/oxidative phosphorylation/complex V	**Atp5f1e**	**ATP Synthase F1 Subunit Beta**
**D**	mito/ATP/oxidative phosphorylation/complex V	**ATP8**	**ATP synthase protein 8**
**D**	mito/ATP/oxidative phosphorylation/complex V	**Tmem70**	**Transmembrane protein 70, mitochondrial**
**D**	mito/ATP/oxidative phosphorylation/complex V	**Atp5h**	**ATP Synthase F1 Subunit Alpha**
**D**	mito/ATP/oxidative phosphorylation/cytochrome c biosynthesis	**Hccs**	**Holocytochrome c-type synthase**
**D**	mito/ATP/oxidative phosphorylation	**Chchd2_1**	**Coiled-coil-helix-coiled-coil-helix domain-containing protein 2**
**D**	mito/ATP/oxidative phosphorylation/predicted	**Mtfr1l**	**Mitochondrial Fission Regulator 1 Like**
**E**	signalling/energy/AMPK	**Prkaa1**	**5'-AMP-activated protein kinase catalytic subunit alpha-2**
**E**	signalling/energy/AMPK	**Prkaa2**	**5'-AMP-activated protein kinase catalytic subunit alpha-2**
**F**	repair/heat stress/hsp	**Hsp70**	**Heat shock 70 kDa protein**
**F**	repair/heat stress/hsp	**Hsp90aa1**	**Heat shock protein HSP 90-alpha**
**F**	repair/heat stress/hsp	**Hsp90ab1**	**Heat shock cognate protein HSP 90-beta**
**F**	repair/heat stress/hsp	**Hspa13**	**Heat shock 70 kDa protein 13**
**F**	repair/heat stress/hsp	**Dnajb4**	**DnaJ heat shock protein family member B4**
**F**	repair/heat stress/hsp	**Dnajc2**	**DnaJ homolog subfamily C member 2**
**G**	repair/Antioxidants	**Gclc**	**Glutamate-cysteine ligase catalytic subunit**
**G**	repair/Antioxidants	**Gpx1**	**Glutathione peroxidase 1**
**G**	repair/Antioxidants	**Prdx6**	**Peroxiredoxin-6**

Journal of Experimental Biology • Supplementary information

**G**	repair/Antioxidants	**Gsta1,Gsta2**	**Glutathione S-transferase alpha-1**
**H**	mito/stress	**Bcap31**	**B Cell Receptor Associated Protein 31**
**H**	mito/stress	**Bnip3l_0**	**BCL2 Interacting Protein 3 Like**
**H**	mito/stress	**Slc25a24**	**Mitochondrial adenyl nucleotide antiporter SLC25A24**
**H**	mito/stress	**Abce1**	**TP-binding cassette sub-family E member 1**

References

Braun, T., Challis, J. R., Newnham, J. P. and Sloboda, D. M. (2013). Early-life glucocorticoid exposure: The hypothalamic-pituitary-adrenal axis, placental function, and longterm disease risk. *Endocr. Rev.* **34**, 885–916.

Casagrande, S. and Hau, M. (2018). Enzymatic antioxidants but not baseline glucocorticoids mediate the reproduction–survival trade-off in a wild bird. *Proc. R. Soc. B Biol. Sci.* **285**,.

Casagrande, S., Stier, A., Monaghan, P., Loveland, J. L., Boner, W., Lupi, S., Trevisi, R. and Hau, M. (2020). Increased glucocorticoid concentrations in early life cause mitochondrial inefficiency and short telomeres. *J. Exp. Biol.* **223**, 222513.

Cooper, C. E., Hurley, L. L., Deviche, P. and Griffith, S. C. (2020). Physiological responses of wild zebra finches (Taeniopygia guttata) to heatwaves. *J. Exp. Biol.* **223**, 1–8.

Dupont, S. M., Grace, J. K., Lourdais, O., Brischoux, F. and Angelier, F. (2019). Slowing down the metabolic engine: Impact of early-life corticosterone exposure on adult metabolism in house sparrows (Passer domesticus). *J. Exp. Biol.* **222**,.

Hau, M., Casagrande, S., Ouyang, J. Q. and Baugh, A. T. (2016). Glucocorticoid-Mediated Phenotypes in Vertebrates: Multilevel Variation and Evolution. *Adv. Study Behav.* **48**, 41–115.

Martin, D. E. and Hall, M. N. (2005). The expanding TOR signaling network. *Curr. Opin. Cell Biol.* **17**, 158–166.

Mentesana, L. and Hau, M. (2022). Glucocorticoids in a warming world : do they help birds to cope with heat waves ? *Horm. Behav.* **142**, 105178.

Picard, M., Juster, R. P. and McEwen, B. S. (2014). Mitochondrial allostatic load puts the “gluc” back in glucocorticoids. *Nat. Rev. Endocrinol.* **10**, 303–310.

Rajagopal, M. C., Brown, J. W., Gelda, D., Valavala, K. V., Wang, H., Llano, D. A., Gillette, R. and Sinha, S. (2019). Transient heat release during induced mitochondrial proton uncoupling. *Commun. Biol.* **2**,.

Rubalcaba, J. G. and Jimeno, B. (2022). Biophysical models unravel associations between glucocorticoids and thermoregulatory costs across avian species. *Funct. Ecol.* **36**, 64–72.

Schradin, C., Makuya, L., Pillay, N. and Rimbach, R. (2023). Harshness is not stress. *Trends Ecol. Evol.* **38**, 224–227.

Stier, A., Romestaing, C., Schull, Q., Lefol, E., Robin, J. P., Roussel, D. and Bize, P. (2017). How to measure mitochondrial function in birds using red blood cells: a case study in the king penguin and perspectives in ecology and evolution. *Methods Ecol. Evol.* **8**, 1172–1182.

Tagliavia, M., Catania, V., Dell'Omo, G. and Massa, B. (2023). High-performance PCR for alleles discrimination of chromo-helicase-DNA binding protein (CHD1) gene in bird sexing. *Biology (Basel).* **12**, 300.

Tilgar, V. (2019). Repeated stimulation of the pituitary–adrenal axis alters offspring phenotype of a wild passerine. *J. Exp. Biol.* **222**, jeb200659.

Valvezan, A. J. and Manning, B. D. (2019). Molecular logic of mTORC1 signalling as a metabolic rheostat. *Nat. Metab.* **1**, 321–333.

Journal of Experimental Biology • Supplementary information
